# Separation of blood microsamples by exploiting sedimentation at the microscale

**DOI:** 10.1038/s41598-018-32314-4

**Published:** 2018-09-20

**Authors:** D. Forchelet, S. Béguin, T. Sajic, N. Bararpour, Z. Pataky, M. Frias, S. Grabherr, M. Augsburger, Y. Liu, M. Charnley, J. Déglon, R. Aebersold, A. Thomas, P. Renaud

**Affiliations:** 1 Microsystems Laboratory (LMIS4), School of Engineering (STI), École Polytechnique Fédérale de Lausanne (EPFL), Lausanne, CH 1015 Switzerland; 2 ARC Training Centre in Biodevices, Faculty of Science, Engineering and Technology, Swinburne University of Technology, Hawthorn, VIC 3122 Australia; 3 Department of Biology, Institute of Molecular Systems Biology, ETH Zurich, Zurich, CH 8093 Switzerland; 4 Unit of Toxicology, CURML, Lausanne University Hospital, Geneva University Hospitals, rue Michel-Servet 1, Geneva, CH 1211 Switzerland; 5 Service of Therapeutic Education for Chronic Diseases, WHO Collaborating Centre, Geneva University Hospitals, University of Geneva, rue Gabrielle-Perret-Gentil 4, Geneva, CH 1205 Switzerland; 6 Division of Laboratory Medicine, Department of Genetics and Laboratory Medicine, Geneva University Hospitals, rue Gabrielle-Perret-Gentil 4, Geneva, CH 1205 Switzerland; 7 Division of Endocrinology, Diabetes, Hypertension and Nutrition, Department of Internal Medicine Specialities, Faculty of Medicine, University of Geneva, rue Gabrielle-Perret-Gentil 4, Geneva, CH 1205 Switzerland; 8 Department of Pharmacology, Cancer Biology Institute, Yale University School of Medicine, West Haven, CT 06516 USA; 9 Centre for Micro-Photonics, Faculty of Science, Engineering and Technology, Swinburne University of Technology, Hawthorn, VIC 3122 Australia; 10 Faculty of Science, University of Zurich, Zurich, CH 8006 Switzerland; 11 Faculty of Biology and Medicine, University of Lausanne, Vulliette 04, Lausanne, CH 1000 Switzerland

## Abstract

Microsample analysis is highly beneficial in blood-based testing where cutting-edge bioanalytical technologies enable the analysis of volumes down to a few tens of microliters. Despite the availability of analytical methods, the difficulty in obtaining high-quality and standardized microsamples at the point of collection remains a major limitation of the process. Here, we detail and model a blood separation principle which exploits discrete viscosity differences caused by blood particle sedimentation in a laminar flow. Based on this phenomenon, we developed a portable capillary-driven microfluidic device that separates blood microsamples collected from finger-pricks and delivers 2 µL of metered serum for bench-top analysis. Flow cytometric analysis demonstrated the high purity of generated microsamples. Proteomic and metabolomic analyses of the microsamples of 283 proteins and 1351 metabolite features was consistent with samples generated via a conventional centrifugation method. These results were confirmed by a clinical study scrutinising 8 blood markers in obese patients.

## Introduction

**Figure 1:**
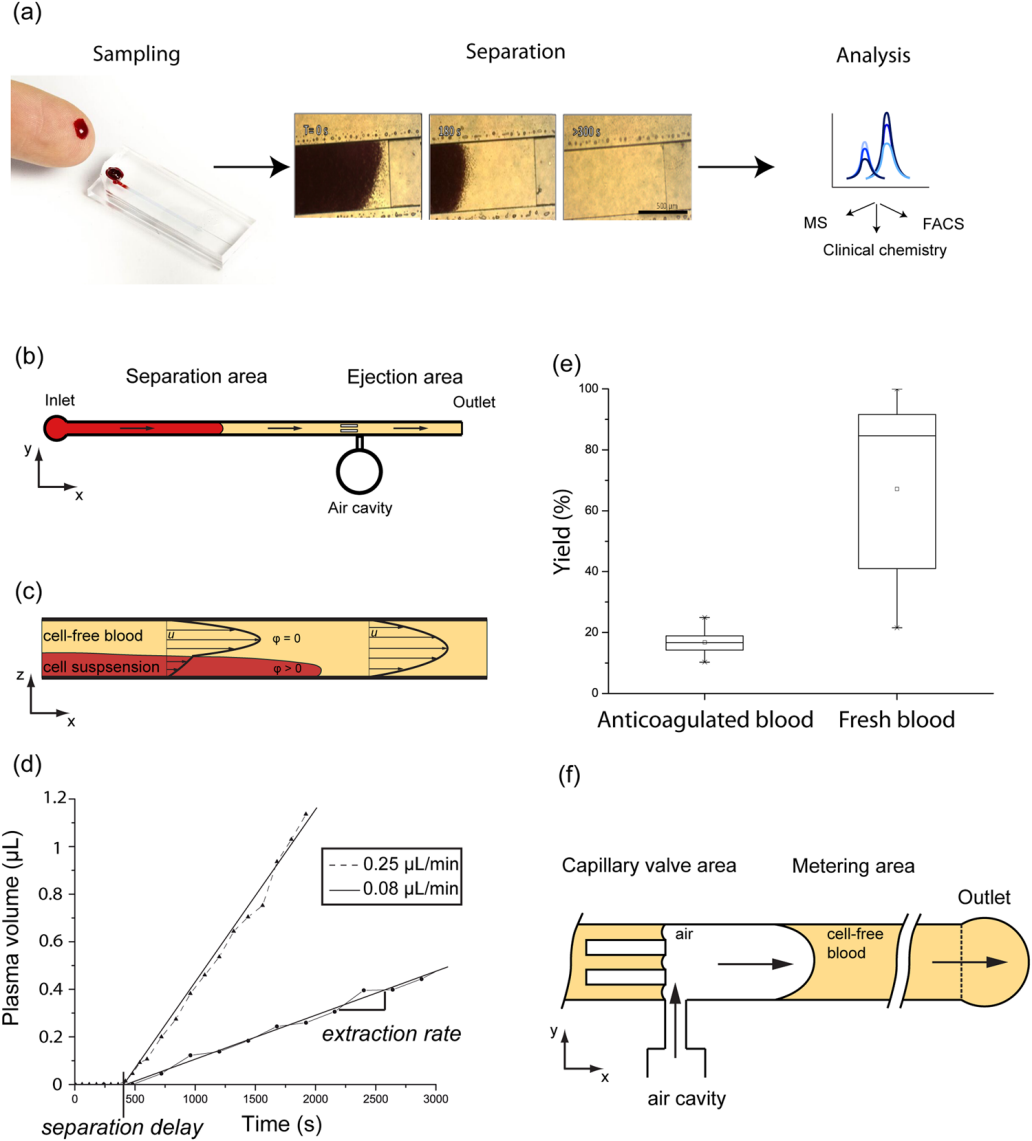
Separation microdevice and fluidic behaviour (**a**) Illustration of the study’s purpose: generation of analytically relevant cell-free blood microsample from fingerprick; (left) sampling of capillary blood after fingerprick and device loading; (center) sequence of microscopic captures depicting the formation of a cell-free plug at the air-liquid interface; (right) retrieval allowing off-chip gold standard analyses; (**b**) Device structure containing two areas performing the main functions: separation and ejection; (**c**) Illustration of the separation principle showing the expected velocity *u* distribution due to the distribution of cellular volume fraction ϕ; (**d**) Typical extracted volume curve in time for 0.08 and 0.25 μl/min whole blood feeding rate; (**e**) Comparison of anticoagulated and fresh sample yields showing a strong increase in fresh blood extraction yield (N = 23 and N = 13 for anti-coagulated and fresh blood extraction yield respectively); (**f**) Ejection mechanism: air injection allows the ejection of a 2 μL liquid sample from the metering area. The volume definition is performed by the capillary valves present in the channel and at the outlet; x, y and z represent the channel longitudinal, transversal and vertical directions respectively.

Blood sample separation, consisting in the extraction and isolation of the liquid surrounding blood cells, is the most common preparation operation performed before clinical biochemistry analysis. With recent advancement of bioanalytical technologies in term of sensitivity and selectivity, the question of the minimal blood volume required for biochemical analyses has become more central, indicating the need for microsampling solutions^1^. Microsampling with typical volumes of 10–100 µL allows less invasive, more frequent and more convenient blood sampling. For preclinical trials on small animals and populations at risk (such as newborns or polymedicated patients), microsample analysis drastically increases subject welfare. Most common microsystems for blood separation traditionally rely on sedimentation^2,3^, microfiltration^4,5^ or cell deviation^6,7^ in a microfluidic chip^8^ (see Supplementary Note 1). However, typically, such systems require either sample pre-dilution, have complex designs or suffer from low extraction yields^9^. Additionally, off-chip sample retrieval is typically not performed as those systems are destined to be integrated in single device as micro total analysis systems (i.e. labs on chip). In this paper we report a device developed for sample preparation at the point of collection (SP-POC) that addresses the need for a blood microsample separation device that generates standardized and stabilized microsamples^10^. The device allows separation of 25 µL undiluted blood samples in a complete passive manner requiring no external equipment: both, separation and pumping are passive. Whole blood is separated into liquid and residual cells using a separation principle based on sedimentation-induced viscosity differences and simultaneous capillary-driven laminar flow. Volume-metered ejection of the separated samples allows off-chip retrieval of 2 µL processed samples for subsequent analysis as illustrated in Fig. 1a. The microfluidic process (modeled in Supplementary Note 2) leads to a low cellular contamination as characterized using flow cytometry. Mass spectrometry proteomics and metabolomics profiling, as well as standard clinical chemistry analysis, were performed on the chip-separated samples to demonstrate their quality and their suitability for gold standard bench-top analysis methods.

## Results

### A passive device for blood separation and volume metering

The device consists of a microfluidic system containing a separation area and an ejection area, as illustrated in Fig. 1b. It is constituted of two polydimethylsiloxane (PDMS) parts assembled together (see Supplementary Fig. 1), one of which is modified with an additive to allow adequate capillary pressure, and effectively triggers spontaneous flow in the device. Each area executes a specific function: separation of blood samples and ejection of a volume-metered output cell-free sample, respectively. While flowing in the separation area cells present in the sample, under the action of gravitational forces, sediment towards the bottom of the channel where neither trenches nor complex structures are present^11^. The formed sediment is at a high volume fraction (ϕ) of cells and is of higher viscosity than the cell-free supernatant^12^ (see Supplementary Note 2 and Supplementary Fig. 2). The simultaneous pumping of the liquid and the generation of the different sediment phases creates a velocity difference between the lower viscosity supernatant and the higher viscosity sediment^13^ as illustrated in Fig. 1c. The extraction of plasma from anticoagulated blood (resp. serum in case of untreated whole blood) starts after a separation delay, necessary to establish through sedimentation a viscosity contrast sufficient to mediate separation as shown in Fig. 1d. The duration of the separation delay is dependent on cell sedimentation speed and distance: the process is thus influenced by blood parameters (e.g. hematocrit, cell size) and design parameters (channel height) but is independent of whole blood feeding flowrate (experimental Pearson’s r = 0.43, N = 20). Upon reaching sufficient height, the clear supernatant starts flowing faster than the sediment and a plug of clear liquid appears at the air-liquid interface: a spatial separation of a plasma (resp. serum) plug occurs along the channel length. This separation along the channel length allows easy manipulation in standard planar technology microfluidic systems. This novel separation mechanism is modeled in Supplementary Note 2 and allows passive separation of whole blood microsamples in a simple device using spontaneous separation phenomena. The separation phenomenon happens in the bulk of the sample and thus allows great flexibility with system design and material choices.

The separation delay, independent of flow rate, was determined to be 400 s ± 148 s (N = 20) and 430 s ± 88 s (N = 13) for anti-coagulated samples and fresh samples respectively (Supplementary Fig. 3). During the subsequent separated liquid extraction phase, the cell-free plug grows continuously as whole blood enters the system: the sediment still progresses in the chip during extraction, however the air-liquid interface progresses at a faster rate. To characterize the separation phenomenon, experiments at constant flow rates were devised with an external fluidic control and anticoagulated blood samples. As shown in Fig. 1d, the extraction rate is constant during the extraction phase and depends on the imposed feeding flowrate. The extraction rate is linearly correlated with the feeding flowrate (adjusted-R2 = 0.96, N = 23) and the yield (see Materials and Methods) achieved is 17% (N = 23; Fig. 1e). The quantity of plasma (respectively serum) separated directly depends on the volume of blood loaded and relates to the maximum respective yields reported. The geometry of the separation and metering channels have an impact on the total separation time, maximum volume that ca be processed, and total volume separated: wider and longer channel designs yield more separated sample volume, and longer channels impose more total separation time. To characterize the fresh blood separation mechanism, the devices were run using the capillary pressure as the driving mechanism. These conditions recreate field operation conditions, where capillary pumping implies non-constant flowrates and clotting drastically changes the sample viscosity. For fresh samples, coagulation leads to a yield increase to 67% (N = 13; Fig. 1e), due to an additional filtration through a formed clot (see Supplementary Note 3).

The integrated sample ejection mechanism illustrated in Fig. 1f allows the off-chip retrieval of the separated sample in a form that is immediately compatible with a variety of downstream gold standard analyses such as flow cytometry, immunoassays or mass spectrometry. The sample volume is restricted to 2 µL by two capillary valves placed on each side of a volume metering area: an in-channel capillary valve and the open outlet. During channel filling, the in-channel capillary valve acts as a delay valve: the liquid front stops an instant upon filling the capillary valve area before spontaneously starting to fill the metering area. Upon complete filling of the metering area, flow in the device stops spontaneously, when reaching the outlet, thereby simplifying sample ejection and increasing the robustness of separation timing. The sample ejection is activated by collapsing a cavity in the chip through external mechanical pressure: the air contained in the cavity (>2 µL) is injected into the channel and drives the liquid movement. At the in-channel capillary valve, an air-liquid interface is created, and surface tension prevents air from traveling towards the inlet. The air drives the liquid content of the metering to the outlet. Thus, the volume ejected precisely corresponds to the volume contained between the in-channel valve and the outlet. The ejection process is completed when the separated sample collected in the metering area is transferred outside the system in the form of a 2 µL drop of separated blood. Excess air is released outside the chip after formation of the drop.

### Sample purity

**Figure 2:**
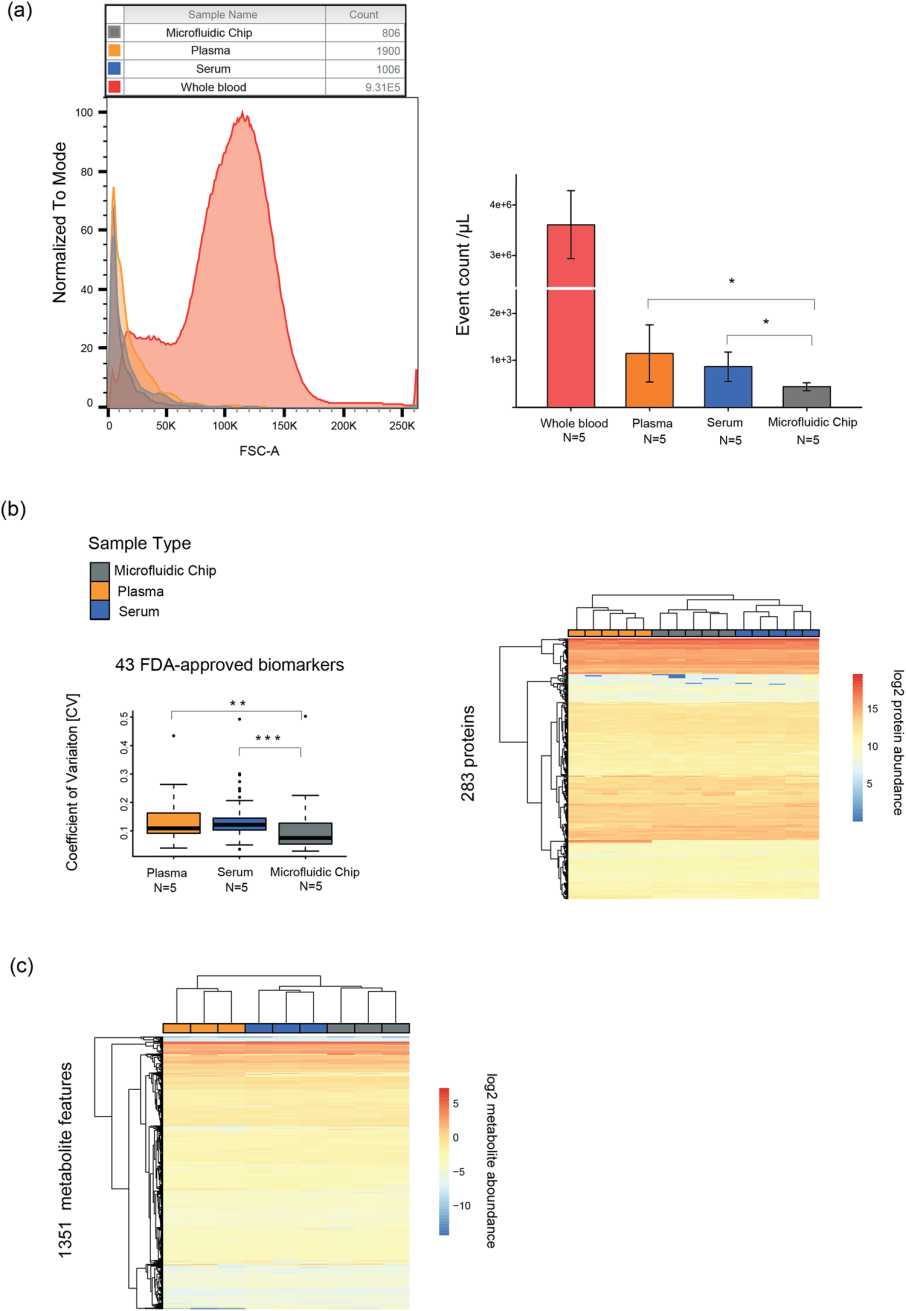
Analytical comparison of three blood separation methods (**a**) Blood samples particle content was analyzed by flow cytometry; (left) normalized event counts vs FSC showing the narrow FSC distribution of events in separated samples vs whole blood; (right) event count per microliter of original samples (N = 5) showing a significantly lower number of particles in chip-separated samples compared to centrifuged plasma or serum samples; (**b**) Proteomic data measured in the capillary blood of healthy volunteer; (left) boxplot presenting coefficient of variations calculated for 43 FDA approved blood biomarkers, in generated cell-free blood samples (N = 5) by plasma, serum or chip-based method (right) non-supervised hierarchical clustering of samples (N = 5) from three blood separation methods based on 283 quantified proteins; (**c**) Metabolomic data measured in the capillary blood of healthy volunteer. Non-supervised hierarchical clustering of samples (N = 3) from three blood separation methods based on 1351 metabolic features. Asterisks indicate statistically significant differences among the tested groups and corresponds to the p-value adjusted for the multiple comparisons: ^*^P = <0.05, ^**^P =  <0.01, ^***^P = <0.001. N corresponds to the number of analytical repetition.

To assess the purity of the chip-separated samples, cell contamination was determined using flow cytometry. Particle size-based distribution was analyzed using forward scattering (FSC) data as shown in Supplementary Fig. 4. Data was compared between device-separated samples and centrifuged samples from capillary whole blood. Typical normalized histograms of FSC values for each sample type is shown in Fig. 2a (left). These results show that the chip-separated samples are marginally contaminated by particles smaller than RBC, represented by the strongly populated range between 75 K and 175 K in the whole blood histograms. As illustrated in Fig. 2a (right), whole blood samples concentration was 3.6·10^6^ particles/µL (coefficient of variation, CV = 17%). For conventional plasma and serum samples, particle concentration was 1.1·10^3^ particles/µL (CV = 48%) and 8.7·10^2^ particles/µL (CV = 32%), respectively. Chip-separated samples contain a significantly lower particle concentration with 4.4 10^2^ particles/µL (CV = 17%) compared to a conventional separation method. Plasma and serum do not yield significantly different particle counts. Purity (see Materials and Methods) achieved for chip-separated samples was 99.987%. In comparison, lower purities were obtained for the reference samples (99.968% for plasma, and 99.976% for serum samples). Thus, this experiment shows that the microdevice separates blood cellular components with a higher repeatability and a higher purity upon comparison with reference samples.

### Biochemical sample content

Liquid blood samples generated with reference methods and chip separation method were compared for proteome content by using a bottom-up proteomic workflow based on SWATH mass spectrometry (SWATH-MS)^14^. For the generation of plasma, serum and chip-separated liquid, whole blood originated from finger-prick capillary sampling on a single donor (young male adult) was used. The generation of liquid samples for each of three blood preparation methods was repeated five times. In total, 15 biological samples were measured, and data metrics were compared between chip-separated liquid, plasma and serum samples. The proteomic analysis identified 312 unique proteins (below 1% of protein FDR) of which 284 proteins were consistently quantified in at least 30% of measured data^15^. Notably, among 284 quantified proteins, 43 have already been approved as plasma biomarkers for various diseases by the Food and Drug Administration (FDA)^16^. To estimate the protein variability between the three different blood separation methods, the coefficients of variation (CVs) of 43 FDA approved proteins were computed. CVs were calculated based on raw protein intensities between five replicates of each method. The protein Beta-2-Microglobulin (B2M) showed generally high variability in all three-sample type. Besides B2M, the coefficient of variation of the FDA biomarkers for conventional plasma and serum data ranged between 3.5 and 30% and did not exceed 22% for the chip-separated liquid (Fig. 2b left). By using one-way ANOVA test, protein variability on chip-separated samples was shown to be significantly lower than for conventionally prepared plasma and serum samples. The differences were statistically significant in both comparisons against plasma (adjusted p-value = 0.003) and also against serum (adjusted p-value < 0.001), with median CV of chip-separated liquid of 7.5% compared to 10.9% and 12.2% for plasma and serum, respectively (Fig. 2b left).

Then, hierarchical cluster analysis was used to group the samples according to the similarity of their quantitative protein profiles. The patterns generated from the 15 blood samples revealed clear division of three different types of blood liquid separations as shown in Fig. 2b (right). Five replicates per sample type were always adjacent to each other, showing an excellent reproducibility for each of the three blood separations. However, two main clusters on the heat-map separated plasma samples from the two closest sub-clusters of serum and chip separated devices. The data indicate the high proteome similarity of chip-separated liquid and conventionally generated blood serum based on the quantitative protein profiles. Remarkably, the subset of proteins that mainly differ in abundance levels in the plasma compared to other serum and chip-separated liquid, were related to blood clotting cascade, such as platelet factors or fibrinogen chains (Supplementary Fig. 5).

The proteomic characterization of the generated samples was confirmed by untargeted metabolomic analyses of the same samples. After matching against known metabolites in the human metabolome database (HMDB, http://www.hmdb.ca) using the mass to charge ratio (*m/z*), the putative metabolite list to interrogate was reduced to 1351 metabolic features, starting from 2 µL of chip-separated samples. Based on quantitative metabolome data, two main clusters on the heat-map distinguished plasma samples from the two closest sub-clusters of serum and chip separated devices (Fig. 2c) with remarkable reproducibility between three repeated samples of respective matrices. Although significant differences of abundance were observed for some metabolites between the three biological matrices, this result confirmed that the chip-separated samples are comparable to serum at the metabolic level. This result also showed that the chip-separated samples would provide similar results than serum or plasma in clinical and biological studies. This is in agreement with previous studies aiming at comparing both plasma and serum matrices for metabolomic investigations^17,18^.

Taken together, proteomic and metabolomic data confirm the high reproducibility of the microfluidic device in generating analytically relevant cell-free liquid from capillary whole blood.

### Diagnostic applications

**Figure 3:**
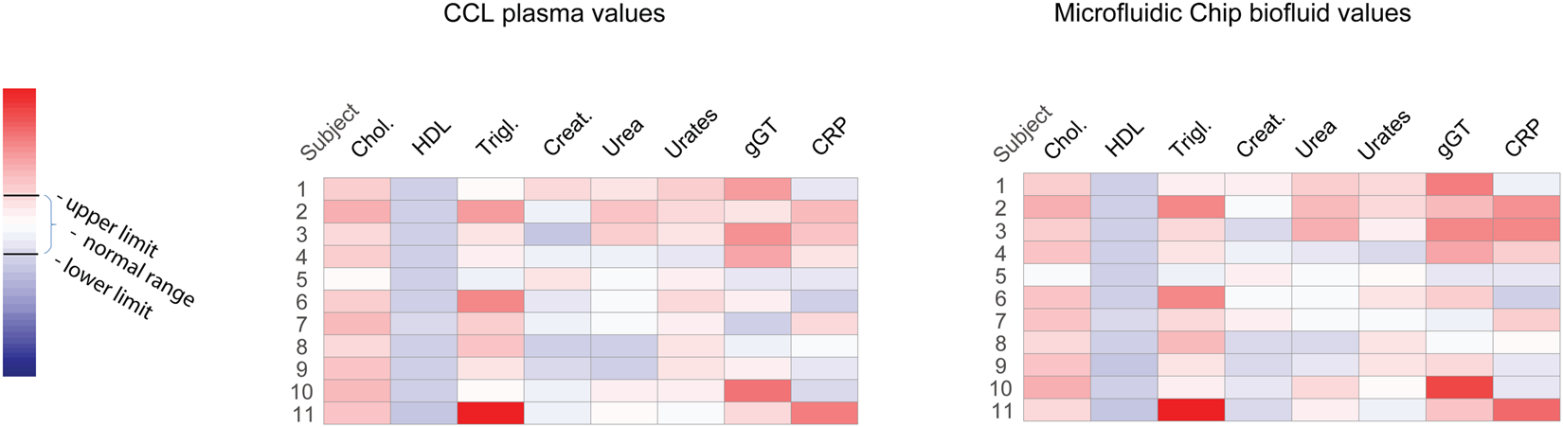
Diagnostic blood parameters. Heatmaps representing abundance of 8 clinical blood markers in 11 obese subjects. Comparison between central clinical laboratory (CCL) plasma values and the chip-separated analytical values. Colors represent values relative to the normal range (see Table 1). Red colors indicate high concentrations, while blue colors represent low concentrations.

The capacity of the microfluidic chip to integrate in a laboratory test cycle was tested on samples collected from 11 obese subjects. Anti-coagulated venous blood was separated in the microfluidic device and diluted prior to being used for standard biochemical analysis. Samples were analyzed on a clinical automated analyser platform that performed 8 typical blood markers for lipidic status, renal and liver functions, and inflammation (see Table 1). In parallel, conventional plasma samples, obtained after centrifugation, were analysed for the same clinical panel in a central laboratory (CCL) and obtained values were used as references. As expected, considering the nature of the subject population, values outside the normal range were found for lipid profile (e.g. triglycerides and HDL) and liver function (e.g. gGT). For each of the eight clinical markers, strong positive linear correlation was observed between chip-separated samples (N = 11) and reference CCL plasma values (Pearson’s r range 0.756 to 0.996) (Table 1). Furthermore, we found strong positive correlations with statistical significance for the 8-panel markers measured between plasma CCL and chip-separated samples for each subject individually (Pearson’s r range 0.991 to 0.999) (see Supplementary Table 1). These results are confirmed by visual inspection of the heatmaps presenting the two tested methods (CCL and microfluidic chip) and show the capacity of the microdevice to integrate in a standard laboratory test cycle (Fig. 3).

**Table 1:** Panel analytes.

Name	System	Normal range	CCL vs chip-separated	
			Pearson r	p-value
Cholesterol total	Lipid profile	<5 mmol/L	0.879	3.71E-4
High Density Lipoprotein (HDL)	Lipid profile	>1 mmol/L	0.918	6.70E-5
Triglycerides	Lipid profile	<1.7 mmol/L	0.996	8.1E-11
Creatinine	Renal function	60–110 µmol/L	0.756	7.13E-3
Urea	Renal function	2.5–7.9 mmol/L	0.969	8.82E-7
Urates	Renal function	160–430 µmol/L	0.960	2.80E-6
γ-Glutamyltransferase (gGT)	Liver Function	10–55 U/L	0.970	8.09E-7
C-Reactive protein(CRP)	Inflammatory state	<10 mg/L	0.977	1.15–6

Analytes in the performed panel and associated systems. Normal range practiced in the Geneva University Hospital for each analyte. Pearson correlations per analyte.

The use of conventional bench-top analytical processes requires substantial dilution factor for overcoming instrument dead volume, thus limiting the panel size upon microsample analysis. The quality of the chip-separated microsamples would allow a larger panel and improved resolution with dedicated low-dead volume analytical protocols and tools.

## Discussion

The SP-POC microfluidic device presented in this work enables the on-site operation-free separation of capillary whole blood microsamples. The quality and reproducibility of the processed samples as well as the off-chip sample retrieval allowed performing robust state-of-the-art bench-top analysis.

The passive separation and the simplicity of the device design allow a strong potential for immediate mass production with high flexibility both in terms of manufacturing methods and materials.

For subsequent bench-top analysis, the SP-POC device could be associated with a sample storage and transport medium to preserve the sample quality from field sampling to high-throughput analysis in a centralized laboratory. In addition, specific lab-on-chip application could also be explored by integrating the microfluidic device described herein into micro total analysis system.

By exploiting the unique microfluidic capabilities of our device for blood separation, this technology could thus bring new insights into all biomedical areas where blood collection and testing is needed and contribute to the active monitoring of diseases and wellness for personalized medicine, potentially impacting millions of end-users.

## Methods

### Fabrication of microfluidic devices

The microfluidic device fabrication is based on a standard soft lithography polydimethylsiloxane (PDMS) process illustrated in Supplementary Fig. 1. The device consists of two parts: the top part contains the imprint of the structure while the bottom is flat and modified with surfactant to obtain an adequate capillary pressure in the device. The top part mold consists of a silicon wafer onto which a 200 μm SU-8 (GM 1070, Gerstelltec, Switzerland) layer is spincoated and photostructured. A 2 mm PDMS (Sylgard 184, Dow Corning, USA) layer is poured on the mold before degassing. The PDMS was prepared from a 1:10 mixture of curing agent and base. The structured PDMS layer is partly cured for 30 min at 80 °C. The bottom part mold consists of a bare silicon wafer exempt of structure. A 1 mm layer of PDMS with the addition of 0.58% of surfactant (Silwet 618, Momentive, USA) is poured on the mold before degassing. The hydrophilic layer is partly cured for 30 min at 80 °C. Both the top and bottom layers are cured simultaneously. Each structured imprint in the top layer is cut to chip size and a 2 mm inlet is punched. The structured layer is put in contact with the hydrophilic part and the chip outlet is created by cutting the channel at its utmost end. The devices are stored at room temperature for at least 24 hours before use. This delay allows for complete polymerization of the materials and the establishment of some adhesion force resulting from cross-linking between the layers.

### Capillary blood collection

All samples used in this work, except those used for clinical chemistry assay, are finger prick capillary blood microsamples. Finger pricks were performed on disinfected skin with contact-activated lancets (Microtainer, BD, USA). The first drop was wiped off to avoid interstitial liquid contamination. The whole blood microsamples were retrieved directly from the skin of the subject using a pipette. The research design and protocol were approved by the Swinburne’s Human Research Ethics Committee (SUHREC), “SHR Project 2016/024 – Extracting and using small volumes of human blood for biomedical device testing”. All methods were performed in accordance with the relevant guidelines and regulation

### Chip-based separation of anticoagulated samples

A 200 μl sample of untreated skin-puncture capillary whole blood was mixed with 1.6% of 2% Coomassie blue water solution for staining and observation convenience and with 0.18 mg/100 μL of dried K2EDTA anticoagulant. The dilution due to the staining is minimal and the anticoagulation doesn’t induce any dilution. The samples are thoroughly mixed before use through tube inversion. A 20 μL sample of prepared blood was loaded in a portion of tubing with the pumping system (Nemesys, Cetoni, Germany). Tubing and syringes were previously filled with water and an inert oil plug (Perfluoromethyldecaline, PFD) to prevent any compliance effect or mixing. The tubing was inserted in the inlet of the device placed in a closed container. The container inner atmosphere was saturated with humidity by using an open water container, thus mitigating evaporation. The pumping system is used during these experiments to impose a constant feeding flow rate. Time lapse imaging with a digital camera (Panasonic, Lumix, Japan) was used to characterize front positions as well as plasma or total flow rate. The chamber’s bottom was lined with millimeter graph paper for dimension calibration purposes. Cellular suspension front and liquid-air front positions were measured, using software (ImageJ), from the center of the inlet through the center line of the channel to the desired front. Extracted volume is determined by computing the volume contained between the cellular front and the liquid-air interface. The infiltration distance is the channel length primed with liquid and it is measured from the inlet center to the liquid-air interface. The feeding rate of whole blood was measured on the time lapse imaging data by measuring the progression of the liquid-air interface, hence the changes in infiltration distance. The plasma extraction rate is computed by extracting the slope of a linear regression of non-zero extracted volume data points; the feeding flow rate was extracted from the linear regression of total fed volume. The yield is computed through ratio between extraction rate and feeding flowrate during the extraction phase. The anticoagulated blood separation delay is computed by using the time axis intercept of the linear regression.

### Chip-based separation of fresh untreated samples

As for anticoagulated samples, a 200 μl sample of untreated capillary whole blood was mixed with 1.6% of 2% Coomassie blue in water for staining and observation convenience. The samples are thoroughly mixed before use through up/down pipetting operations. A 25 μL of prepared blood was loaded with a pipette in the chip inlet port. This quantity is in excess, as the chip total volume is 7 μL. The sample totally fills the inlet and creates a drop on the chips top part. Liquid flows in the system through sole capillary action. The chips were, prior to loading, placed in a closed container with high humidity to mitigate evaporation during the experiments. Time lapse imaging with a digital camera (Panasonic, Lumix, Japan) was used to characterize front positions. Yield and separation delay are computed on still images as no linear regression can be applied if capillary filling is used. The cell-free liquid extraction yield is computed by measuring, in the time-lapse imaging, the total volume loaded during the extraction phase in the chip main channel and the total generated cell-free liquid. This value represents a time average yield during the complete extraction phase. The fresh blood separation delay is determined by identifying the first time point with a resolvable clear plug in the time lapse imaging data.

### Reference separated sample generation

Separated samples were generated as references for analysis: plasma and serum samples. Plasma samples were generated from fresh whole blood collected in EDTA-coated tubes and centrifuged for 10 min at 2000 RCF. Plasma aliquots were retrieved through supernatant pipetting. To generate serum samples, fresh whole blood was allowed to clot for 15 min at ambient condition before being centrifuged for 10 min at 2000 RCF. Serum aliquots were retrieved through supernatant pipetting.

### Flow cytometry analysis

Flow cytometry (BD FACSAriaTMIII, BD Biosciences, USA) was used to determine cell counts and size distribution in raw blood and separated samples. Four sample of different nature were characterized in this comparison: whole blood, plasma, serum and chip-separated liquid from untreated whole blood. The samples originated from the same subject and are all retrieved through skin puncture. For whole blood samples, 2 μL of fresh whole blood were sampled from the surface of the subject punctured skin. The samples were immediately diluted in 300 μL of Phosphate Buffered Saline (PBS). 30 μL of this sample was further diluted 10x in PBS. For plasma samples, 150 μL of fresh whole blood were anticoagulated before being centrifuged for 10 min at 2000RCF. A 2 μL aliquot of the resulting plasma was then diluted into 300 μL of PBS. For serum samples, 250 μL of fresh whole blood were allowed to clot for 15 min at ambient condition before being centrifuged for 10 min at 2000 RCF. A 2 μL aliquot of the resulting serum was then diluted into 300 μL of PBS. For the chip-separated samples, each chip was loaded with a fresh 25 μL sample of untreated fresh whole blood. The 2 μL ejected from the chip were recovered with a pipette before being diluted into 300 μL of PBS.

### Proteomic analysis

Three different blood preparation protocols were characterized for proteomic content. Equal amount of 2 µL of plasma, serum or chip-separated liquid was used for overnight trypsin digestion of single samples. In total we digested 15 samples, five replicates per each blood preparation protocol. Next day, peptide digests were cleaned on MACROSpin Plate-VydacSilicaC18 (Nest Group Inc., USA), solubilized in 50 μL of 0.1% aqueous formic acid (FA) with 2% acetonitrile (ACN) and were used for final MS analysis.

### SWATH assay library

We used publicly available SWATH assay library^19^ previously generated on TripleTOF 5600 mass spectrometer equipped with a NanoSpray III source and heated interface (AB Sciex, Canada) from depleted plasma digest fractionated by strong anion exchanger (SAX) and full non-fractionated plasma samples^19^.

### SWATH-MS Measurement and Data analysis

15 blood samples were measured on TripleTOF 5600 mass spectrometer operated in SWATH mode as described earlier^14^. Reverse phase peptide separation was performed with linear nanoLC gradient as described before. An accumulation time of 100 ms was used for 64 fragment ion spectra of 12.5 m/z each and for the precursor scans (SWATHs) acquired at the beginning of each cycle, resulting in a total cycle time of 3.3 s. The SWATHs were overlapping by 1 m/z and thus cover a range of 400–1200 m/z. Raw SWATH data files were converted into the mzXML format using ProteoWizard (version 3.0.3316)^20^ and data analysis was performed using the OpenSWATH tool^15^ integrated in the iPortal workflow^21^.

The recorded feature intensities after OpenSWATH identification were filtered through SWATH2stats^22^ to reduce the size of the output data and remove low quality features. This resulted in a list of 312 proteins achieving protein FDR below 1% in all the samples measured.

Then these filtered fragment intensities were analysed by the R/Bioconductor package MSstats (version MSstats.daily 2.3.5) and converted to relative protein abundances that were used for further statistical data analysis^23^.

### Metabolic analyses

Similar to proteomic analysis, three different blood preparation protocols were characterized for proteomic content. Equal amount (2 µL) of serum, plasma or chip-separated samples were extracted using 50 µL of mix solvent EtOH:MetOH:H2O in the ratio of 2:2:1. Overall, 9 samples were extracted, three replications for each subtype of blood samples. All samples were then vortexed mixed for 30 s, incubated for 10 min at 4 °C and centrifuged for 10 min at 14000 rpm and 4 °C. The supernatants were removed and evaporated to dryness using speed vacuum concentrator (SpeedVac SPD1010, Thermo Fisher Scientific, USA) and stored at −80 °C until analysis.

Untargeted metabolomic analysis was performed on UPLC system (Dionex, Thermo Fisher Scientific, USA) hyphenated with HRMS, hybrid quadrupole-Orbitrap mass spectrometer (Q Exactive, Thermo Fisher Scientific, USA). HRMS was interfaced with an electrospray ionization (ESI) source. Data acquisition was done in both negative (NEG) and positive (POS) polarities using similar setting: sheath gas flow rate 40, auxiliary gas flow rate 10, capillary temperature 320 °C, S-lens RF 50 and auxiliary gas heater temperature 300 °C. The sweep voltage was optimized for each ionization mode to have the proper spray current (POS: 3.3 kV and NEG: 2.9 kV). Moreover, lock mass was considered with respect to the acquisition mode which permit real-time recalibration by correcting m/z shifts due to instrumental drift. Reverse phase chromatography was done using C18 Kinetex, 2.6 μm, 50 mm × 2.1 mm I.D. column (Phenomenex, USA). Mobile phase was composed of A = 0.1% Formic acid in H_2_O and B = 0.1% Formic acid in MeOH. We used identical mobile phase for both positive and negative ionization modes. Elution was carried out in gradient condition with the mobile phase composition changed from 98% A (0–6 min) to 100% B (6–9 min). To keep system reproducibility, we considered 3 min for system re-equilibrating. The overall run time was roughly 13 min. Flow rate was set at 0.3 mL/min and the injection volume was 3 μL.

Raw data was converted to the mzXML format using ProteoWizard MsConvert version 3.0.7331. The mzXML files were then processed using open-source freely available software XCMS online for peak detection, chromatogram alignment and isotope annotation^24^. This process provided alignment of drift (retention time and accurate mass) in data and ensured that a chromatographic signal (i.e., metabolite feature (*m/z* x RT X intensity)) is identified with the same parameters in each sample^25^. To reduce data complexity, a home-made script was used to remove background by filtering data matrix according to a predefined *m/z* list of metabolites. This list, including around five thousand of present and/or detected metabolites in biofluids, is built from the human metabolome database (HMDB, http://www.hmdb.ca) and updated form our own library.

### Statistical data analysis for proteomics and metabolomics

Using R package “pheatmap” on the log-transformed, normalized relative protein and metabolite intensities, hierarchical data clustering analysis was performed to generate two-dimensional centered heat map. Manhattan distance as distance measure was used for clustering of scaled data. Specifically for proteomic analysis, the coefficient of variation between five repetitions of each protocol were calculated on the normalized raw protein intensities for the 43 detected FDA biomarkers. Box plots were generated in Rstudio (version 3.0.2) by using the ggplot2 package.

### Clinical chemistry

This study, approved by the local ethics committee, was based on 11 informed subjects who declared their consent. Fasted subjects were taken two successive samples of whole blood through a single venipuncture. The first samples were processed in the Central clinical laboratory (CCL) of the Geneva University Hospitals, where usual plasma separation and clinical automated analyser (Cobas 8000, Roche Diagnostics, Switzerland) testing was performed. The second group of samples was taken in k2EDTA tubes (Vacutainer, BD, USA). From these tubes, plasma was generated within 7 hours of sampling both through the microfluidic separation device and through standard centrifugation protocol. During the interval, they were continuously mixed on roller mixers. Prior to experiments, the samples were mixed by inverting the tube at least 3 times and vortexing. For microfluidic separation purposes, a 10 μL blood microsample was loaded in a portion of tubing with the pumping system (Nemesys, Cetoni, Germany). Tubing and syringes were priory filled with water and a PFD oil plug to prevent any compliance effect or dilution through mixing. The tubing was inserted in the inlet of the 5 devices operated simultaneously and the devices were placed in a closed Petri dish with an open water container. The chip pumping operation protocol was the following: (i) load inlet until front is visible in the chip at 100 μl/min (ii) loading of 2 µL at 10 μl/min (iii) separation at 0.1 μl/min (0.5 mm/min linear speed) until cellular front neared the valve. The second operation was performed to ensure that blood entering the channel would not have undergone sedimentation in the inlet. Using the device with anticoagulated blood yields approximately 1 μL per device. The liquid recovered from the outlet was transferred and merged in a container and total volume (of approximately 5 μL) was determined through weighing. The chip-separated samples were diluted in 100 μL (approximately 1:20 dilution) of 0.9% NaCl solution before use. The final results were corrected to account for the precise dilution ratio obtained. This dilution allowed obtaining sufficient sample volume for the operation of analysis. Analysis was performed through spectrophotometric techniques on an automated analyzer (AU480, Beckman-Coulter, USA)

## Electronic supplementary material

Supplementary Material
