# Supplementary material for: Separation of blood microsamples by exploiting sedimentation at the microscale
Source: Sci Rep. 2018 Sep 20;8:14101. doi: 10.1038/s41598-018-32314-4 (PMC6147834; doi:10.1038/s41598-018-32314-4)
Supplement: Supplementary file 1 — Supplementary Material [file 41598_2018_32314_MOESM1_ESM.pdf]

Supplementary material: Separation of blood microsamples by exploiting sedimentation at the microscale

D. Forchelet[#*1], S. Béguin[*2], T. Sajic[3], N. Bararpour[4], Z. Pataky[5], M. Frias[6,7], S. Grabherr[4], M. Augsburger[4], Y. Liu[3,8], M. Charnley[9], J. Déglon[4], R. Aebersold[3,10], A. Thomas[‡4,11] and P. Renaud[‡1]

[1] Microsystems Laboratory (LMIS4), School of Engineering (STI), École Polytechnique Fédérale de Lausanne (EPFL), Lausanne, CH-1015, Switzerland
[2] ARC Training Centre in Biodevices, Faculty of Science, Engineering and Technology, Swinburne University of Technology, Hawthorn, VIC 3122, Australia
[3] Department of Biology, Institute of Molecular Systems Biology, ETH Zurich, Zurich, CH-8093, Switzerland
[4] Unit of Toxicology, CURML, Lausanne University Hospital, Geneva University Hospitals, rue Michel-Servet 1, Geneva, CH-1211, Switzerland
[5] Service of Therapeutic Education for Chronic Diseases, WHO Collaborating Centre, Geneva University Hospitals, University of Geneva, rue Gabrielle-Perret-Gentil 4, Geneva, CH-1205, Switzerland
[6] Division of Laboratory Medicine, Department of Genetics and Laboratory Medicine, Geneva University Hospitals, rue Gabrielle-Perret-Gentil 4, Geneva, CH-1205, Switzerland
[7] Division of Endocrinology, Diabetes, Hypertension and Nutrition, Department of Internal Medicine Specialities, Faculty of Medicine, University of Geneva, rue Gabrielle-Perret-Gentil 4, Geneva, CH-1205, Switzerland
[8] Department of Pharmacology, Cancer Biology Institute, Yale University School of Medicine, West Haven, CT 06516, USA
[9] Centre for Micro-Photonics, Faculty of Science, Engineering and Technology, Swinburne University of Technology, Hawthorn, VIC 3122, Australia
[10] Faculty of Science, University of Zurich, Zurich, CH-8006, Switzerland
[11] Faculty of Biology and Medicine, University of Lausanne, Vulliette 04, Lausanne, CH-1000, Switzerland

*Co-first authors
‡Co-last authors
# Correspondence to david.forchelet@epfl.ch

Content

Supplementary note 1 : State of the Art.. 3
Supplementary note 2 : Blood stratified flow.. 7
Supplementary note 3 : Coagulation 12
Supplementary Figures.. 14
Supplementary Tables.. 20
Supplementary References 21

Supplementary note 1 : State of the Art

Microfluidic systems have been developed for blood processing since the inception of the domain. Analytical systems, bearing a few exceptions, require off-chip sample preparation and lack any integration of this process. Lab-on-chip (LOC) approaches for plasma/serum separation have been proposed since the mid-2000s. These approaches are predominately aimed towards microsamples however some high throughput continuous methods could be used for bigger volumes or continuous applications. A list of available methods will be presented here, commenting on their adaptability to microsample volumes. In such blood separation devices, performance is usually characterized around two numbers: purity and yield (see Materials and Methods).

A main division between methods can be elaborated on whether there is an external field applied by the device or not: they are active or passive devices respectively. Passive devices, sometimes referred as “fluidic-only”, rely only on spontaneously occurring phenomena in a flowing liquid at microscale. In passive methods, cells are separated through physical properties such as size, deformability, density, etc. Sedimentation, even though gravity is an external force field, is considered to be a passive method as no external force field is applied by the device. Active methods use an electrical, magnetic, acoustic or centrifugal external force to separate cells from their surrounding liquid. These separation methods rely usually on properties such as dielectric, magnetic or mechanical properties. If the separation method is described as passive or active, the pumping mechanism could be also described as such, depending on the method for liquid movement. The devices are separated in pumpless and pump actuated devices. *Capillary-driven* is used to describe pumpless devices where surface tension forces generate the driving forces for the liquid motion.

Sedimentation is a phenomenon that is used commonly in hematology as a test where erythrocyte sedimentation rate is measured. Under the action of gravity, RBCs settle towards the bottom of the container. They form a highly concentrated layer on the container floor: the sediment. This spontaneous separation is used in different implementation in microfluidic systems either alone or combined with filtration for increase of performance[1]. A common characteristic for most microfluidic systems based on sedimentation is to use multilevel structures to take advantage of the vertical separation of the different components of blood[2–4]. However, the throughput and operation times are limited by the sedimentation speed of RBCs.

Filtration is an obvious method to retain and separate cells in blood samples. The filter uses pores defined in the structures to geometrically exclude cellular components bigger than the pore size from the flow of liquid. Two configurations have been reported: *dead-end* where the cell-free liquid is extracted in the direction of incoming cell suspension[5–7] and *cross-flow* where the cell-free liquid is extracted perpendicularly to the incoming cell flow[8–10]. If flow rates can be high, in general, clogging is the main limiting factor especially in dead-end filtration configurations. Clogging typically leads to low yields in such systems. Due to the viscosities and the fluidic resistances at play, pressures used are high and thus can generate cell leakage through the filter and hemolysis.

Cells flowing in microfluidic systems are subjected to forces that create a deviation of the cells and deplete them from certain regions of the flow. Separated blood is recovered from the cell

depleted flow regions. There exists a variety of phenomenon that are taking advantage of to segregate particles: from Deterministic Lateral Displacement (DLD), Fahraeus effect to Dean vortices. DLD devices consist of a pillar array in which flow around the individual pillar purifies layers of blood flow[11,12]. The cell-free layers are retrieved while the cell concentrated samples are discarded. At low Reynolds number, in microchannels, viscous lift forces tend to push cells towards the center of the channel and create a cell-free liquid layer against the wall: the Farhaeus effect. This cell-free layer is usually collected by creating a bifurcation and taking advantage of Zweifach-Fung effect[13–15]. In bent or curved channels, in flow regimes where Re>1, vortices – called Dean vortices - appear perpendicularly to the global flow rate direction. In addition to inertial forces, particles are subjected to these vortices and new equilibrium positions are generated that allow the retrieval, through bifurcations, of separated samples[16,17]. Yield and purity are usually a tradeoff in all those cell deviation systems. If those systems are not necessarily suited for extraction of blood from microsamples, the strong flow rate used in these methods (especially when operated at Re>1) makes them good candidates for separation of sample of volumes in the order of magnitude of milliliters or for continuous separations.

If microfluidic systems described in this note are traditionally based on a channel configuration, paper-based devices are making a strong impact in the microfluidic applications. If lateral flow assays are common, other devices have been recently developed on the paper support[18–21]. Paper-based devices performing plasma separation rely not only on the paper matrix for performing filtration but also for being the matrix where liquid flow occurs and pumping the liquid through capillary forces. Flow behavior can be manipulated by the addition of specific backing layers. In general, paper-based devices for separation offer a low yield and low purity plasma; however, the ease of integration with other paper-based detection method is a clear advantage of these methods.

The technologies presented here offer different characteristics summarized in

Table 1:1. There is no universally more efficient method: depending on the requirements in terms of throughput, available sample volume, yield, purity or tolerance to hemolysis, a different method would be more adequate. As an example, for continuous separation, cell deviation techniques are the most adequate due to the high flow rates in the systems. However, these methods require a complex system and a skilled worker to dilute the samples and operate the system.

Table 1:1 : Performance comparison of passive microfluidic separation methods

Methods	Advantage	Disadvantage	Sample
Sedimentation	Low hemolysis<br>No precise flow rate required<br>No dilution<br>High purity<br>Potentially high yield	Time efficiency might be low<br>Extraction rate low	Microsample
Microfiltration	High extraction rate	Clogging and low yield<br>Hemolysis and low purity with pressure<br>Membrane surface affinity with analytes<br>Often dilution to reduce clogging/cell leakage	Microsample
Cell deviation	High extraction rate<br>Continuous operation<br>Flexible designs	Dilution needed<br>Precise external pumping	Continuous
Paper devices	Simple and cost effective	No sample retrieval<br>Limited testing<br>Hemolysis<br>Chromatographic effect<br>Adsorption of molecules of interest	Microsample

Supplementary note 2 : Blood stratified flow

Blood is a suspension containing a high volume fraction of cells. Those cells form the solid fraction: the dispersed phase, whereas the plasma forms the liquid fraction of blood: the dispersed medium. In a suspension, volume density of the particles and volume density of the dispersion media are typically different. Thus, due to buoyancy and gravity forces, particles are subject to a total net force. Depending on the volume densities, particles move along gravity towards the bottom of their container or against gravity towards the top of the container. Most commonly, particles settle following gravity: they sediment. Against a physical barrier such as the container bottom, particles create highly concentrated layers.

The viscosity of a particle suspension depends on the concentration of particles in the dispersion medium[22]. For suspensions with a low volume fraction of particles, the viscosity increase is mainly due to viscous drag upon particle movement in the fluid [23]. When concentrations reach high values, such as in the case of blood, particle-particle hindering mechanisms dominate[24]. **Erreur ! Source du renvoi introuvable.** describes the viscosity of a highly concentrated suspension in Newtonian conditions[25]. This model was shown to fit blood's behavior over a range of hematocrit (equivalent to volume fraction).

$$\mu = \mu_0 \left(1 - \frac{\varphi}{\varphi_{max}} \right)^{-2} \quad (1)$$

where μ is the effective viscosity of the suspension, μ_0 is viscosity of the dispersion medium, φ is the volume fraction, φ_{max} is the maximal packing fraction.

Blood flowing in horizontal tubing at moderate velocity, where mixing is negligible, has been observed to form what was described as layered flow[26]. The structure is described as a 2 phase system: a clear cell-free plasma layer above a concentrated cell suspension layer. The difference in hematocrit amongst layers, generated by the sedimentation process, create a difference in viscosity. The cell-free supernatant is less viscous than the sediment. Upon application of pressure, the velocity profile is influenced by the local variations in viscosity as shown in Figure 2:1. Pressure here can be generated by external equipment or capillary forces.

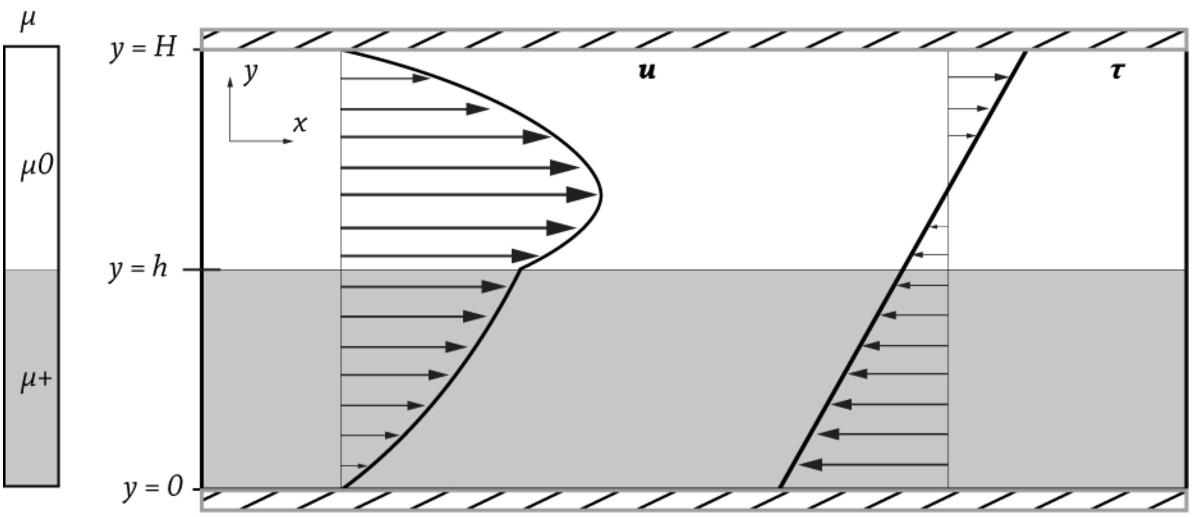

Figure 2:1 : Illustration of the velocity field (u) and shear stress τ for a stratified Poiseuille flow. H denotes the channel height. Phase viscosities are µ0 and µ+ for the upper and lower layers respectively

Considering a channel of height H initially filled with blood at hematocrit (or volume fraction) φ_i, if the sediment-clear supernatant interface is placed at height h, the sediment hematocrit φ_+ is given by

$$\varphi_+ = \frac{H}{H-h}\varphi_i \quad (2)$$

Considering the liquids as Newtonian, Equation 3 applies. Under incompressible laminar flow conditions, the momentum equilibrium is given by Cauchy Equation 4.

$$\tau = \mu \frac{du}{dy} \quad (3)$$

$$\frac{\partial \rho \boldsymbol{u}}{\partial t} + \rho \boldsymbol{u} \cdot \nabla \boldsymbol{u} = -\nabla P + \nabla \cdot (\boldsymbol{\tau}) \rightarrow \frac{d\tau}{dy} = -\frac{dP}{dx} \quad (4)$$

where τ is the shear stress, ρ is the volume density, t is the time, P is the pressure, x is the channel longitudinal coordinate, y is the channel vertical coordinate.

These equations are valid in each layer. However, they are invalid at the interface due to the viscosity discontinuity.

The boundary conditions in such a flow in channels are typical of Poiseuille flow and presented in Equation 5. They stem from the no slip conditions.

$$u(0) = 0, u(H) = 0 \quad (5)$$

At the interface and because of the no slip conditions, the continuity of both the velocity and the shear stress are guaranteed.

$$u \in C^0, \tau \in C^1 \tag{6}$$

Solving analytically this system yields a velocity profile given by Equation 7.

$$u(y) = \begin{cases} \dfrac{1}{\mu_0}\dfrac{dP}{dx}(y^2 - H^2) + \dfrac{\dfrac{dP}{dx}h^2 + \dfrac{\mu_+}{\mu_0}\dfrac{dP}{dx}(h^2 - H^2)}{\mu_0 h - \mu_+(h - H)}(y - H)\ for\ y\ \in [h, H] \\ \dfrac{1}{\mu_+}\dfrac{dP}{dx}y^2 + \dfrac{-\dfrac{1}{\mu_0}\dfrac{dP}{dx}h^2 + \dfrac{1}{\mu_0}\dfrac{dP}{dx}(h^2 - H^2) + \dfrac{\dfrac{dP}{dx}h^2 + \dfrac{\mu_0}{\mu_+}\dfrac{dP}{dx}(h^2 - H^2)}{\mu_+ h - \mu_0(h - H)}(h - H)}{h}\ for\ y\ \in [0, h] \end{cases} \tag{7}$$

The flow rate in each layer is given by :

$$Q_{top} = \int_h^H u(s)ds\ ,\ Q_{bottom} = \int_0^h u(s)ds \tag{8}$$

where Q_{top} is the supernatant flow rate and Q_{bottom} is the sediment flow rate.

The parameters used in the following calculations are reported in Table 2:1. The channel height is a fixed design parameter. The sediment thickness was determined through observations to be approximately 2/3 of the channel height. This value corresponds with reported configurations[27]. The plasma viscosity, dependent on temperature, is adjusted for room temperature.

Table 2:1: Computation parameters

Parameter	Symbol	Value	Note and reference
Channel height	H	200 µm	Channel height
Sediment thickness	h	134 µm	Experimental observations and previous work[27]
Plasma viscosity	μ_0	1.8 mPa·s	For 20○C. Considering 1.25 mPa·s at 37○C [22]
Max packing fraction	φ_{max}	0.75	In the interval 0.73-0.8 [28]
Initial hematocrit	φ_i	0.45	Physiological range 0.4-0.54 for men [29]

Considering the sediment thickness and a homogeneous sediment, the sediment hematocrit is computed to be 67%. This creates a strong viscosity contrast: sediment layers are approximately 88 times more viscous than supernatant. Computed flow velocity profile is reported in Figure 2:2. It can be observed that most of supernatant flows faster than the sediment, i.e. the average speed of the supernatant is noticeably higher than that of the sediment. Even though the supernatant thickness is half that of the sediment, the strong viscosity contrast implies that the supernatant flow rate is 128% that of the sediment.

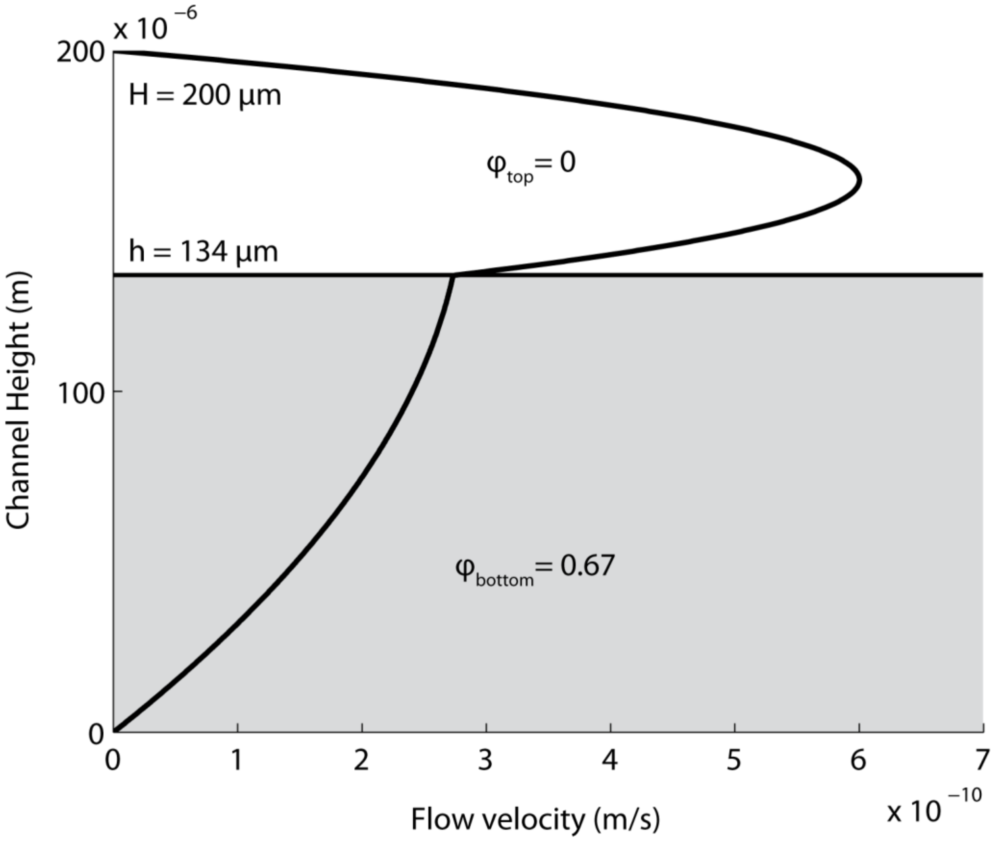

Figure 2:2: Resulting velocity profile of a sediment occupying 2/3rd of the separation channel height.

In the system discussed here, the retrieved top layer volume flows downstream in the channel compared to the bottom thicker layer. The yield is modeled in Equation 9 by the ratio between this downstream volume of supernatant to overall volume entering the system.

$$Y = \frac{\frac{Q_{top}}{Q_{bottom}} - \left(\frac{H}{h} - 1\right)}{\frac{Q_{top}}{Q_{bottom}} + 1} \quad (9)$$

Using reported values, the yield is computed to be 34%. This value corresponds roughly to measurements reported in this work.

In the two-layer time independent model presented here, effect of sedimentation dynamics has not been accounted for. The establishment of the different layers has an important impact on the separation as shown by the discussed separation delay. The height of the interface between layers is also expected to be time-dependent as it is directly affected by sedimentation

times: in this model it is fixed to a value given by experimental observation. The sediment is supposed to be homogeneous and concentration gradients in the sediment itself are neglected.

Supplementary note 3 : Coagulation

Prevention of blood loss, *hemostasis*, consists of several mechanisms amongst which platelet agglutination and blood coagulation are central ones. Small holes in vessel walls are often sealed by a loose platelet plug. This plug is densified during the subsequent blood coagulation. Blood coagulation or clotting is a mechanism that, through several steps, leads to the conversion of fibrinogen to fibrin fibers. Those fibers form a meshwork that entraps blood cells and adheres to damaged vessel walls. The liquid that is extracted from the clot, either through natural retraction or through centrifugation, is called serum and is exempt of fibrinogen and other clotting factors. It thus differs from plasma which contains those components.

A key difference between separation of anticoagulated and fresh samples is the clotting that occurs. During the first instants of extraction, the separation mechanism is assumed to be similar between anti-coagulated and fresh samples: plasma is extracted. Then in the case of fresh blood, coagulation starts to occur and the biofluid being generated transitions from pure plasma to pure serum. The nature of the final sample is therefore expected to be a combination of a majority of serum and a minority of plasma.

Separation phenomenon has proven to be very stable for anti-coagulated samples, whereas fresh samples exhibit a variable increase of yield with time. The clotting of the dense sediment probably creates a plug that filters the blood: serum is extracted from the clot towards the plug. The high yield number show that, for most devices, blood entering the channel is filtered upon entry: thus concentrating blood cells in the inlet and loading the channel with separated liquid. Fresh blood extraction yield (in Figure 1) is thus noticeably higher than expected plasma proportion in the sample. The variation in clotting dynamics explains the high variation shown in the yield. Coagulation is expected to increase variability due to its intrinsic patient-dependent and time-dependent nature[30]. This filtration mechanism is likely to be responsible for higher separation yields. Albeit any filtration mechanism could theoretically increase hemolysis in the sample, no hemolysis has been observed in the device presented in this paper.

Supplementary Figure 5 represents the intensity of peptides associated to the fibrinogen chains. Fibrinogen signals intensity is noticeably lower in chip-separated and serum samples compared to plasma values. The fibrinogen levels are not significantly dissimilar between chip and serum for α-chain (adj. p-value = 0.705, t-test) and β –chain (adj. p-value = 0.199, t-test), while they are significantly dissimilar for the γ-chain (adj. p-value = 0.033, t-test). Thus, this experiment shows that the chip-separated liquid has undergone coagulation. If the chip-separated sample resembles strongly serum, some minor coagulation component differences are still present between the chip-separated samples and the serum. Such a result seems to indicate that the sample could be a mixture of large quantities of serum with small quantities of plasma: this is coherent with previous experimental observations.

The absence of platelets from the flow cytometry experiments is also a consequence of the coagulation of fresh samples in the chip: at least 99.85% of platelets are rejected. This rejection is much higher than predicted by the simple sedimentation: individual platelets sedimentation rate is approximately only 55 nm/s. Considering platelets as individual cells is representative of their behavior in the blood flow; however, upon blood sampling, platelets start to participate to the hemostasis. Platelets aggregate by adhering to each other[31] : thus having a much bigger hydrodynamic radius and sedimenting at higher speeds. Also, platelets have a tendency to

adhere to channel walls where shear rate is maximal[32] and to fibrin clots[33]. As shown in previous experiments, coagulation occurs in the microdevice; it is thus expected that platelets constitute part of the clot and are thereby strongly depleted from the separated liquid.

Supplementary Figures

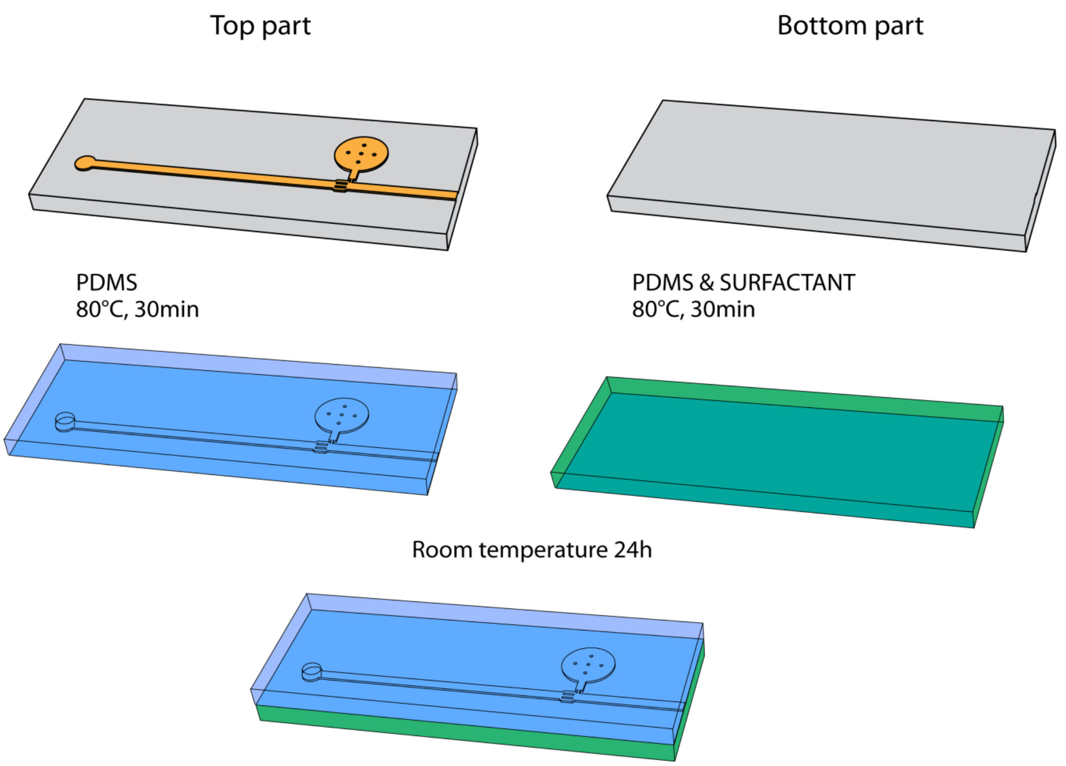

Supplementary Figure 1: Device fabrication process based on a PDMS soft lithography process. Top part is made of bare PDMS while bottom part contains an additive to generate the necessary capillary effect.

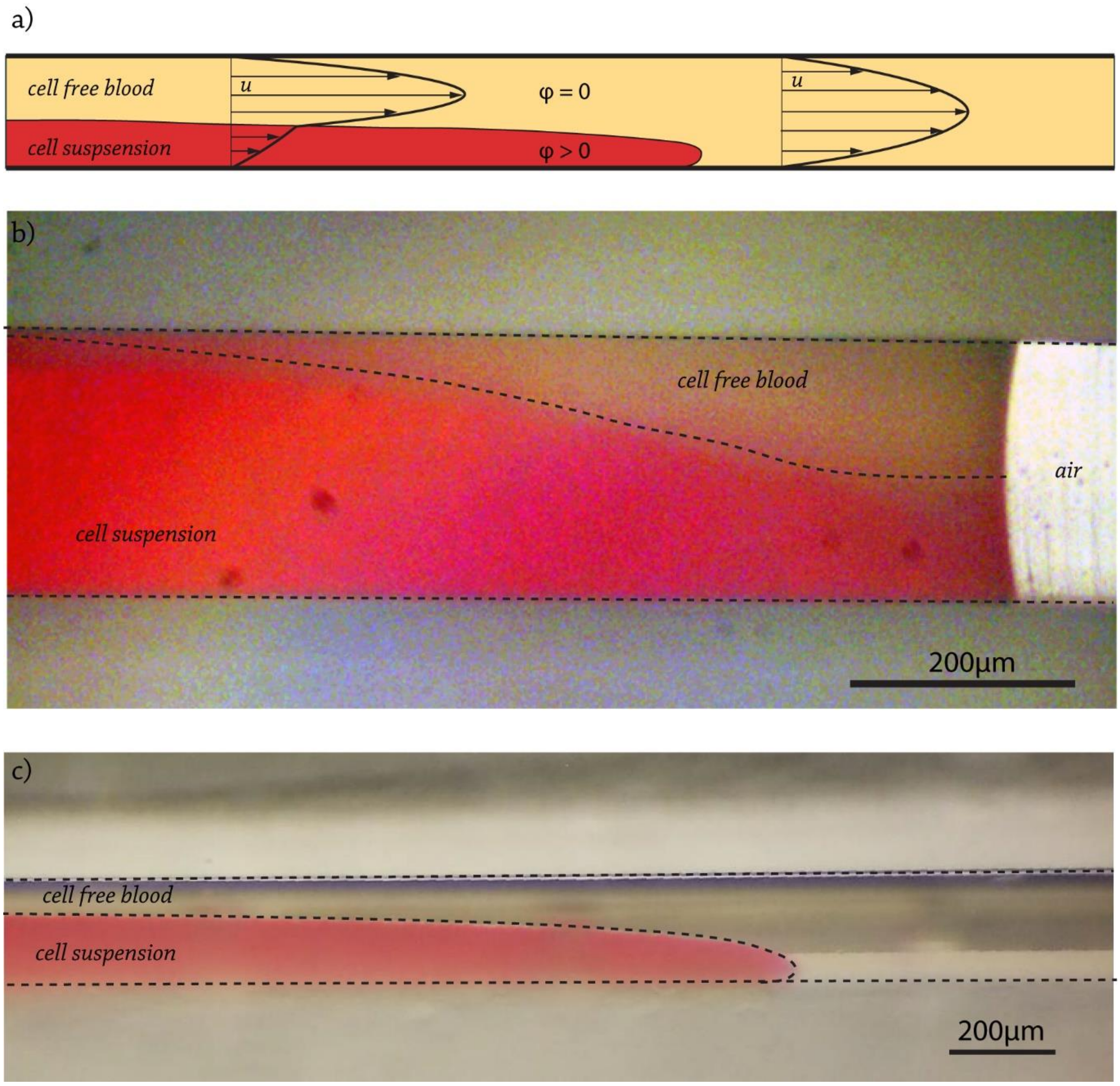

Supplementary Figure 2: Side views of the sedimentation-based separation (a) Illustration of the separation principle showing the expected velocity *u* distribution (b) Front with cell suspension before separation delay. The cell-free supernatant layer is forming but the plug has not formed yet (c) Cellular front during extraction phase showing the clear separation between sediment and supernatant

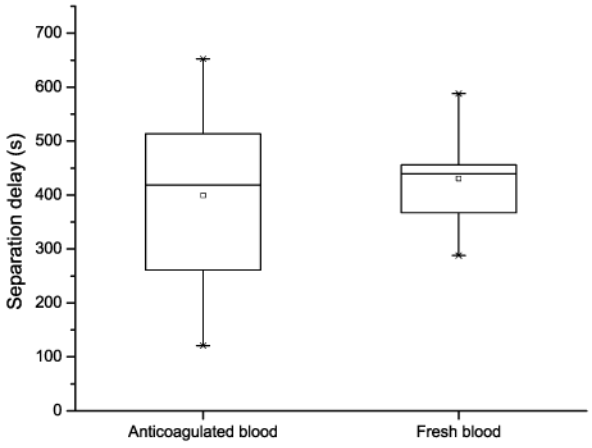

Supplementary Figure 3: Comparison of anticoagulated and fresh sample separation delay. The separation delay show a strong similarity for both sample types

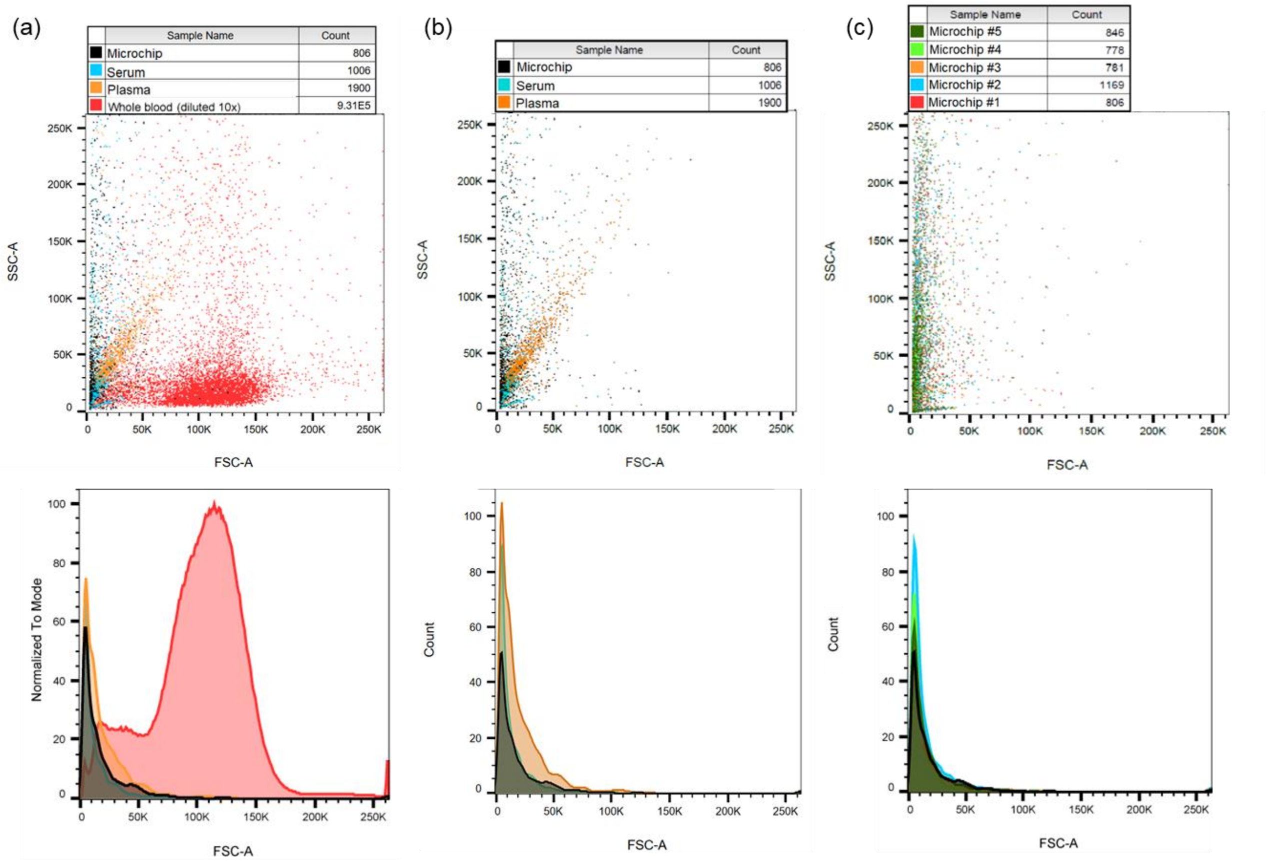

Supplementary Figure 4: Blood samples particle content analyzed by flow cytometry. 1st row: SSC vs FSC scatter plot showing complexity vs relative size of particles in corresponding samples. Associated event counts are presented in the legend. 2nd row: raw and normalized event counts vs FSC showing the narrow FSC distribution of events in separated samples vs whole blood. (a) Samples generated by microchip vs plasma and serum matrices vs whole blood diluted 10x. (b) Typical results for separated matrices. (c) Replicates of microchip-separated samples.

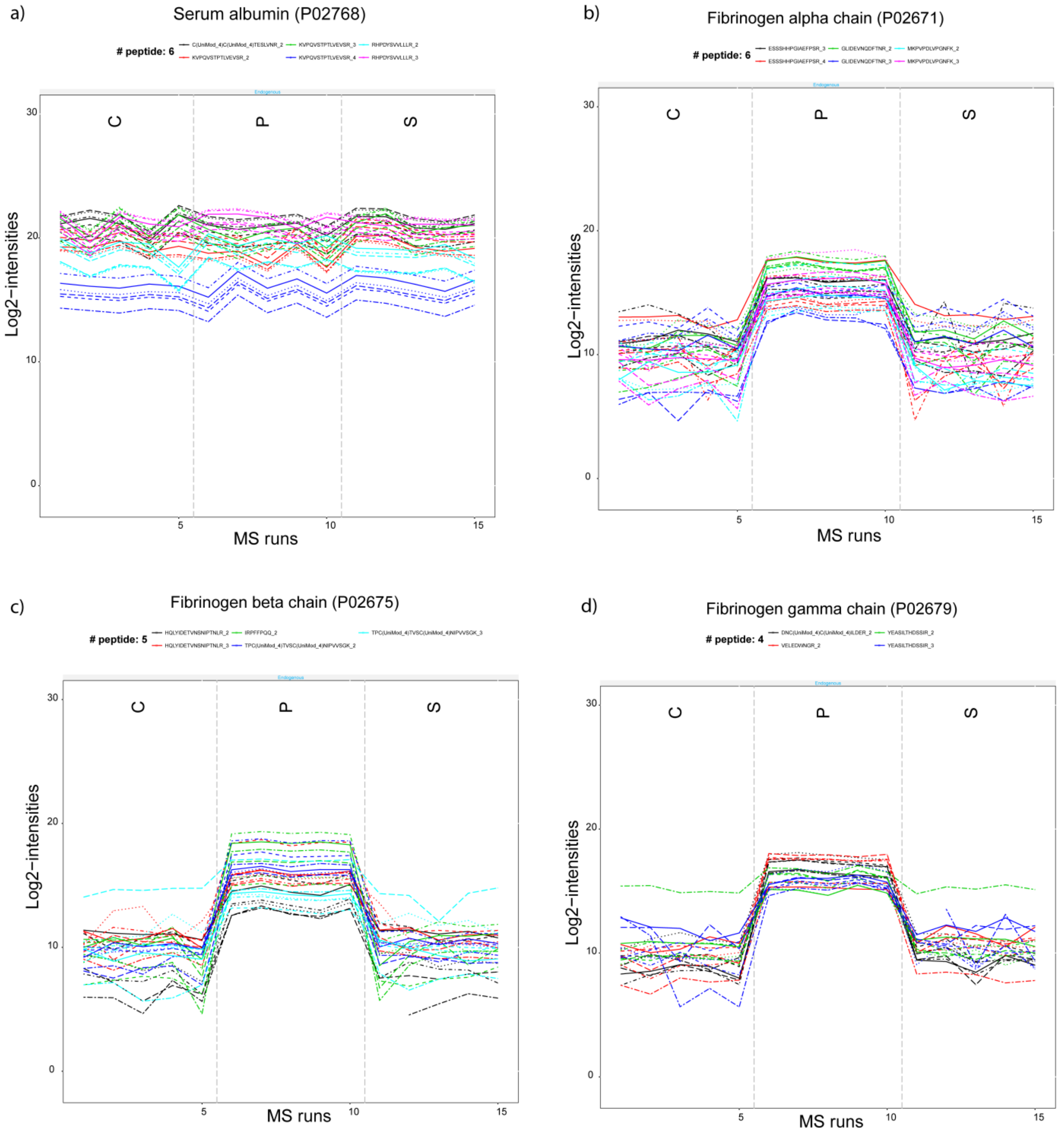

Supplementary Figure 5: Signal intensity for the peptides associated with 4 selected proteins. Each color group represents a peptide resulting from the enzymatic digestion of the protein. Each line style in a color group corresponds to a different transition. Five samples of each matrix (C: chip-separated, P: plasma, S: serum) are run. a) albumin-associated peptide levels showing the constancy of albumin concentration across analytical matrices. b) fibrinogen α-associated peptide levels showing the reduction in level of this coagulation protein for chip-separated and serum samples. c) fibrinogen β-associated peptide levels showing a similar reduction d) fibrinogen γ-associated peptide levels showing a similar reduction

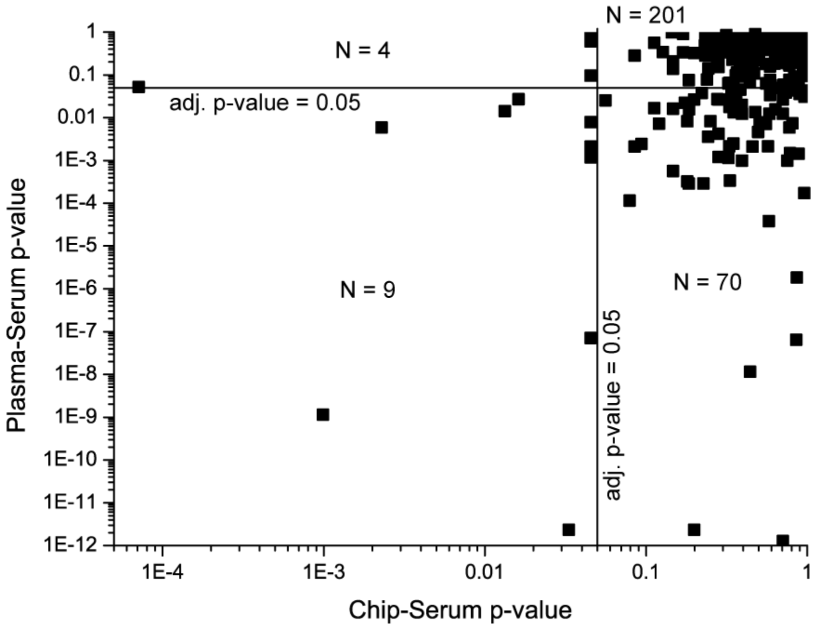

Supplementary Figure 6: T-test statistical test results for 284 protein levels. Comparison of FDR adjusted p-value for chip-separated and serum samples and p-value for plasma and serum samples. 13 proteins show a significant dissimilar level between chip-separated and serum samples. The wide majority of proteins (95.4%) did not exhibit any significant difference in quantity between chip-separated and serum samples: amongst which, proteins known for their surface fouling properties such as α-2-macroglobulin. In comparison, between plasma and serum samples, 79 protein levels are significantly different. 9 of these 13 proteins also show a significant dissimilarity in levels between plasma and serum. The majority of these 9 proteins, such as fibrinogen-γ and platelet factors, are implicated in the coagulation process. Thus showing that the chip-separated sample is similar to serum mixed with small quantites of plasma.

Supplementary Tables

Supplementary Table 1: Pearson correlations of testing methods based on the 8-panel markers values per patient

Subject	CCL vs chip-separated	
	Pearson r	p-value
Subject 1	0.998	4.01E-9
Subject 2	0.998	6.26E-9
Subject 3	0.999	5.18E-10
Subject 4	0.998	2.81E-7
Subject 5	0.999	6.30E-10
Subject 6	0.998	2.35E-7
Subject 7	0.991	1.50E-6
Subject 8	0.995	1.68E-7
Subject 9	0.992	8.91E-7
Subject 10	0.994	3.69E-7
Subject 11	0.998	4.42E-9

Supplementary References

1. Tachi, T., Kaji, N., Tokeshi, M. & Baba, Y. Simultaneous Separation , Metering , and Dilution of Plasma from Human Whole Blood in a Microfluidic System Simultaneous Separation , Metering , and Dilution of Plasma from Human Whole Blood in a Microfluidic. *Anal. Chem.* **81,** 3194–3198 (2009).

2. Dimov, I. K. *et al.* Stand-alone self-powered integrated microfluidic blood analysis system (SIMBAS). *Lab Chip* **11,** 845–850 (2011).

3. Samborski, A. *et al.* Blood diagnostics using sedimentation to extract plasma on a fully integrated point-of-care microfluidic system. *Eng. Life Sci.* **15,** 333–339 (2015).

4. Zhang, X. B. *et al.* Gravitational sedimentation induced blood delamination for continuous plasma separation on a microfluidics chip. *Anal. Chem.* **84,** 3780–3786 (2012).

5. Homsy, A. *et al.* Development and validation of a low cost blood filtration element separating plasma from undiluted whole blood. *Biomicrofluidics* **6,** 12804 (2012).

6. Thorslund, S., Klett, O., Nikolajeff, F., Markides, K. & Bergquist, J. A hybrid poly(dimethylsiloxane) microsystem for on-chip whole blood filtration optimized for steroid screening. *Biomed. Microdevices* **8,** 73–79 (2006).

7. Wang, S. *et al.* Simple filter microchip for rapid separation of plasma and viruses from whole blood. *Int. J. Nanomedicine* **7,** 5019–28 (2012).

8. Crowley, T. A. & Pizziconi, V. Isolation of plasma from whole blood using planar microfilters for lab-on-a-chip applications. *Lab Chip* **5,** 922–929 (2005).

9. Chen, X., Cui, D. F., Liu, C. C. & Li, H. Microfluidic chip for blood cell separation and collection based on crossflow filtration. *Sensors Actuators, B Chem.* **130,** 216–221 (2008).

10. Sollier, E., Cubizolles, M., Faivre, M., Fouillet, Y. & Achard, J. L. A passive microfluidic device for plasma extraction from whole human blood. *Proc. 31st Annu. Int. Conf. IEEE Eng. Med. Biol. Soc. Eng. Futur. Biomed. EMBC 2009* **141,** 7030–7033 (2009).

11. Huang, L. R. Continuous Particle Separation Through Deterministic Lateral Displacement. *Science (80-.).* **304,** 987–990 (2004).

12. Davis, J. a *et al.* Deterministic hydrodynamics: taking blood apart. *Proc. Natl. Acad. Sci. U. S. A.* **103,** 14779–14784 (2006).

13. Faivre, M., Abkarian, M., Bickraj, K. & Stone, H. Geometrical focusing of cells in a microfluidic device: an approach to separate blood plasma. *Biorheology* **43,** 147–159 (2006).

14. Yang, S., Ündar, A. & Zahn, J. D. A microfluidic device for continuous, real time blood plasma separation. *Lab Chip* **6,** 871–880 (2006).

15. Fan, R. *et al.* Integrated barcode chips for rapid, multiplexed analysis of proteins in microliter quantities of blood. *Nat. Biotechnol.* **26,** 1373–1378 (2008).

16. Di Carlo, D. Inertial microfluidics. *Lab Chip* **9,** 3038–3046 (2009).

17. Blattert, C. *et al.* Microfluidic blood/plasma separation unit based on microchannel bend

structures. *2005 3rd IEEE/EMBS Spec. Top. Conf. Microtechnology Med. Biol.* **2005,** 38–41 (2005).

18. Martinez, A. W., Phillips, S. T., Butte, M. J. & Whitesides, G. M. Patterned paper as a platform for inexpensive, low-volume, portable bioassays. *Angew. Chem. Int. Ed. Engl.* **46,** 1318–20 (2007).

19. Martinez, A. W. *et al.* Programmable diagnostic devices made from paper and tape. *Lab Chip* **10,** 2499–504 (2010).

20. Yang, X., Forouzan, O., Brown, T. P. & Shevkoplyas, S. S. Integrated separation of blood plasma from whole blood for microfluidic paper-based analytical devices. *Lab Chip* **12,** 274–280 (2012).

21. Songjaroen, T., Dungchai, W., Chailapakul, O., Henry, C. S. & Laiwattanapaisal, W. Blood separation on microfluidic paper-based analytical devices. *Lab Chip* **12,** 3392–3398 (2012).

22. Yilmaz, F. & Gundogdu, M. Y. A critical review on blood flow in large arteries; relevance to blood rheology, viscosity models, and physiologic conditions. *Korea Aust. Rheol. J.* **20,** 197–211 (2008).

23. Einstein, A. Berichtigung zu meiner Arbeit: „Eine neue Bestimmung der Moleküldimensionen". *Ann. Phys.* **339,** 591–592 (1911).

24. Krieger, I. M. & Dougherty, T. J. A Mechanism for Non-Newtonian Flow in Suspensions of Rigid Spheres. *Trans. Soc. Rheol.* **3,** 137–152 (1959).

25. Quemada, D. Rheology of concentrated disperse systems and minimum energy dissipation principle - I. Viscosity-concentration relationship. *Rheol. Acta* **16,** 82–94 (1977).

26. Schmid-Schönbein, H. Fahraeus-effect-reversal (FER) in compaction stasis (CS): microrheological and haemodynamic consequences of intravascular sedimentation of red cell aggregates. *Biorheology* **25,** 355–366 (1988).

27. Murata, T. Effects of sedimentation of small red blood cell aggregates on blood flow in narrow horizontal tubes. *Biorheology* **33,** 267–283 (1996).

28. Sartory, W. K. Prediction of Concentration Profiles during Erythrocyte sedimentation by hindered settling model. *Biorheology* **11,** 253–264 (1974).

29. H.Billett, H. Hemoglobin and hematocrit. *Clin. Methods Hist. Phys. Lab. Exam. 3rd Ed.* 718–719 (1990). doi:10.1055/s-2004-821156

30. Mann, K. G., Orfeo, T., Butenas, S., Undas, A. & Brummel-Ziedins, K. Blood coagulation dynamics in haemostasis. *Hamostaseologie* **29,** 7–16 (2009).

31. Born, G. V. R. & Cross, M. J. The Aggregation of Blood Platelets. *J. Physiol.* **168,** 178–195 (1963).

32. Gutierrez, E. *et al.* Microfluidic devices for studies of shear-dependent platelet adhesion. *Lab Chip* **8,** 1486–1495 (2008).

33. Hoffman, M. Remodeling the blood coagulation cascade. *J. Thromb. Thrombolysis* **16,** 17–20 (2003).
